# Prevalence and risk factors of asthma, rhinoconjunctivitis and eczema in the very extreme environment of Naryn, Kyrgyzstan^☆^

**DOI:** 10.1016/j.waojou.2026.101386

**Published:** 2026-05-01

**Authors:** Shaiirbek A. Sulaimanov, A. Elena Martinez-Torres, Luis Garcia-Marcos, Mukhtar E. Asheraliev, Artur A. Moidunov, Nurlan N. Brimkulov, Zhanara K. Muratova, Saltanat E. Omusheva, Kubanych T. Tynybekov, Baktybek E. Narkoziev

**Affiliations:** a National Center for Maternity and Childhood Care, Bishkek, Kyrgyz Republic; b Paediatric Allergy and Pulmonology Units and Nurse Research Group, Virgen de la Arrixaca University Children's Hospital, and IMIB Bio-health Research Institute, Murcia, Spain; c Paediatric Allergy and Pulmonology Units, Virgen de la Arrixaca University Children's Hospital, University of Murcia, and IMIB Bio-medical Research Institute, Murcia, Spain; d Department of Research and Management I. K. Akhunbaev, Kyrgyz State Medical Academy, Bishkek, Kyrgyz Republic; e Osh Interregional Children's Clinical Hospital, Osh, Kyrgyz Republic

**Keywords:** Asthma, Rhinoconjunctivitis, Eczema, Prevalence, Kyrgyzstan, ISAAC, GAN

## Abstract

**Background:**

There is not much information about the prevalence of allergic diseases in high altitudes in central Asia; and even less on their potential risk of protective factors.

**Methods:**

A survey using the Global Asthma Network (GAN) methods was carried out in adolescents 13–14 years of age in Naryn (Kyrgyzstan), a small population with a cold, high-altitude, strongly continental climate. GAN questionnaires were self-administered in the school setting and height and weight measured in standardized way. The association between potential risk factors and the different disease indicators, as defined in previous GAN repots was expressed as crude or adjusted odds ratios after logistic regression analyses.

**Results:**

A total of 2673 were included in the analyses (participation rate, 93%). Indicators of asthma were 3.1%, 1.7% and 2.8% for current symptoms, severe current symptoms and asthma ever, respectively. The corresponding figures for rhinoconjunctivitis and eczema were: 13.3%, 1.7%, 16.0%; and 2.9%, 0.9%, 4.9%. Consistent factors associated with at least 1 indicator of asthma, rhinoconjunctivitis, or eczema were: increased physical activity, frequent paracetamol use, and low olive oil intake. Higher burger intake and higher BMI were associated only with current rhinoconjunctivitis. Truck traffic was associated only with eczema ever.

**Conclusions:**

The prevalence of disease indicators of asthma, rhinoconjunctivitis, and eczema in Naryn are among the lowest in the world. The positive association between exercise and most allergy indicators, and the inverse association between olive oil intake and asthma, deserve further attention.

## Introduction

The prevalence of asthma, rhinoconjunctivitis, and eczema in many countries and centres within countries has been quite recently reported by the Global Asthma Network (GAN)^1^ in 2 different age groups (adolescents 13–14 years old and children 6–7 years of age). One of the main findings of the report was the significantly lower prevalence of asthma among countries with medium-low- and low per capita income according to the World Bank as compared with those of high income. According to this organisation, Kyrgyzstan (Kyrgyz Republic) had a per capita income of 2420.2 USD in 2024^2^ which qualify it for the group of lower-middle income countries (1136–4495 USD). GAN included centres of the region with similar income such as Tyumen in Russia; Karaj and Yazd in Iran; and Damascus and Lattakia in Syria. However, risk factors at a centre or country level have not been yet reported.

Furthermore, Naryn (the administrative centre of Naryn Region) is one of the most mountainous areas of Kyrgyzstan and lies at an altitude of about 2000 m. The climate is very harsh: in winter, temperatures may drop to −40°C, and the average winter temperature is around −17°C.^3^ This extreme environment is not shared by any other centre in GAN Phase I survey.

The analysis of the prevalence and risk factors of asthma, rhinoconjunctivitis and eczema under those extreme conditions might allow to add new information on the epidemiology of asthma, further check that in this area the prevalence is lower than in others and add information about the risk factors which can be operating there, and which might be different to those which are important in other geographical areas, either related to the income level, environment or local traditions.

Additionally, the cross-sectional information of the present study can be compared to a previous study performed in other centres in Kyrgyzstan (Jalal Abad, Bishkek and Balykchy), which was carried out within the Phase Three of the International Study of Asthma and Allergies in Childhood (ISAAC)^4^^,^^5^ almost 25 year previously.

## Methods

The present survey was carried out following the GAN Phase One methodology, which follows that of ISAAC, and which has been previously published.^6^

### Questionnaires of symptoms

The definitions of symptoms were extracted from the written questionnaires completed by adolescents (13–14 years of age) while in the school classroom. The original questionnaire was in English and translation and back-translation to Russian and Kyrgyz followed a specific methodology common to ISAAC and GAN.^7^^,^^8^

### Questionnaire administration

After granting permission from the regional and Naryn city health and education authorities, written questionnaires were administered while adolescents were at school, in their preferred language, following the GAN guidelines.^9^

### Definitions

Following previous definitions,^1^ symptoms of asthma, rhinoconjunctivitis, and eczema were classified as follows:

#### Asthma symptoms

“Current asthma symptoms” where defined by a positive answer to the question, “Have you had wheezing or whistling in the chest in the past 12 months?” in the written questionnaire. “Current severe asthma symptoms” was defined as those with current asthma symptoms who, in the past 12 months, and as stated in the questionnaire, have had ≥4 attacks of wheeze, or >1 night per week sleep disturbance from wheeze, or wheeze affecting speech. ‘‘Asthma ever’’ was defined as a positive answer to the question, ‘‘Have you ever had asthma?’’

#### Rhinoconjunctivitis symptoms

“Current rhinoconjunctivitis symptoms” was defined from the affirmative answers to two different questions in the written questionnaire: “In the past 12 months, have you had a problem with sneezing, or a runny or blocked nose when you did not have a cold or the flu?” and “In the past 12 months, has this nose problem been accompanied with itchy-watery eyes” “Symptoms of severe rhinoconjunctivitis” was defined by the response “a lot” to the question “In the past 12 months, how much did this nose problem interfere with your daily activities? (Not at all, a little, a moderate amount, a lot)”. “Hay fever ever” was defined as a positive answer to: “Have you ever had hay fever?”

#### Eczema symptoms

“Current eczema symptoms” was defined as a positive answer to those 2 questions: “Have you had this itchy rash (defined in a previous question) at any time in the past 12 months?” and “Has this itchy rash at any time affected any of the following places: the folds of the elbows, behind the knees, in front of the ankles, under the buttocks, or around the neck, ears or eyes?”. “Severe current eczema symptoms” was defined as current symptoms being the cause of awakening 1 or more times per week after the question “In the past 12 months, how often on average, has this child been keep awake at night by this itchy rash? (Never in the past 12 months, less than 1 night per week, 1 or more nights per week). “Eczema ever” was defined as a positive answer to the question “Have you ever had eczema?”.

### Environmental questionnaire

Following the GAN environmental questionnaire,^8^ the following potential risk or protective factors were analysed: sex, vigorous exercise in 1 week (never or occasionally, once or twice per week, 3 or more times per week); hours/day of television watching (less than 1 h, 1 h but less than 3 h, 3 h but less than 5 h, 5 h or more) hours/day of computer use (less than 1 h, 1 h but less than 3 h, 3 h but less than 5 h, 5 h or more); number of older and younger siblings; frequency of truck traffic near home (never; seldom -not often-, frequently through the day, almost the whole day); paracetamol consumption in the past 12 months (never, at least once a year, at least once per month); cat or dog at home during the last 12 months (yes, not); tobacco smoking at any time (not at all; less than daily; daily); tobacco smoking in the last 12 months (not at all; less than daily; daily); age of starting smoking; number of cigarettes/day; and water pipe smoking (yes, no).

Additionally, the monthly consumption of different foods (never or only occasionally, once or twice per week, most or all days) was analysed. Those foods included: meat, seafood, fruit, cooked vegetables, raw vegetables, pulses, cereals, bread, pasta, rice, margarin, butter, olive oil, milk, other dairy products, eggs, nuts, potato, sugar, burger, other fast food, and soft drinks.

### Height and weight measurements

Height and weight were measured at school by fieldworkers in a standardised way by means of stadiometer and scale (Medical Stadiometer MSC-234, China).

### Data handling and analysis

Data was recorded in paper questionnaires. The obtained data were typed in an Excel sheet according to the data entry instructions of the GAN manual.^8^ The obtained dataset was then sent to the GAN Data Centre in Murcia for its validation, with the same algorithms as those used with the official GAN centres, to clean it, check the data in the electronic form against that on paper when discrepancies arose, and make it comparable with the whole GAN Phase One dataset.

The convention to admit participation rates of datasets were the same as in ISAAC: at least 80% for the adolescents. For participation calculations, the denominator was the number of children in the age group cluster sample, and the numerator the number of core questionnaires returned with at least 1 data symptom. Por prevalence estimations, positive answers to a specific symptom in the centre was divided by the number of completed questionnaires.

Venn diagrams for the prevalence and overlapping of disease indicators were performed through DeepVenn.^10^ The association between potential risk factors and the different disease indicators was expressed as crude or adjusted odds ratios. Adjusted odds ratios (expressed as OR (95%CI) were obtained by multiple logistic regression analyses including all factors yielding significant (p < 0.05) or near significant (0.05 < p < 0.15) crude odds ratios. Calculations were made by means of Stata/SE V19 (College Station, TX, USA).

Two sets of multiple logistic regression analyses were performed: the first one (adjusted model, aOR) including all the associated factors (p < 0.15) found in the bivariate analysis; and the other (fully adjusted model, faOR) including those factors together with the corresponding disease indicators not used as the dependent variable. For example, for “current asthma symptoms” (dependent variable) adjustment was made including all significant associated factors together with “current rhinoconjunctivitis symptoms” and “current eczema symptoms”.

## Results

A total of 2673 adolescents were included in the analysis (participation rate, 93%). Table 1 shows the prevalence of the different disease indicators for asthma, rhinoconjunctivitis and eczema with the 95% CI. Indicators of asthma were 3.1%, 1.7% and 2.8% for current symptoms, severe current symptoms and asthma ever, respectively. The corresponding figures for rhinoconjunctivitis and eczema were: 13.3%, 1.7%, 16.0%; and 2.9%, 0.9%, 4.9%.

**Table 1:** Prevalence of disease indicators in the population (N = 2673)

	n	%	95%CI
Indicators of asthma:			
Current asthma	83	3.1	2.5–3.8
Severe asthma	46	1.7	1.3–2.3
Asthma ever	76	2.8	2.3–3.5
Indicators of rhinoconjunctivitis:			
Current rhinoconjunctivtis	356	13.3	12.1–14.7
Severe rhinoconjunctivitis	44	1.7	1.2–2.2
Hay fever ever	428	16.0	14.7–17.5
Indicators of eczema:			
Current eczema	130	4.9	4.1–5.7
Severe eczema	23	0.9	0.6–1.3
Eczema ever	115	4.3	3.6–5.1

The proportion of potential risk or protective factors in the population is shown in web Table 1 (environmental) and 2 (diet). There was a very low proportion of smoking (either cigarettes or water pipe) among the adolescents included in the survey with only 0.3% of them admitting to currently smoke cigarettes daily and 2.3% less than daily. Water pipe smoking was also very infrequent (0.5%) (web Table 1). It is also of interest the high proportion of individuals not doing any vigorous exercise during a week (71.2%).

**Fig. 1:**
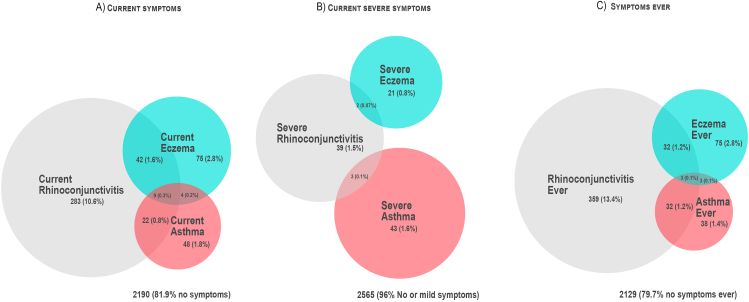
Overall prevalence of unique and overlapping disease indicators. (A) Current symptoms. (B) Severe current symptoms. (C) Symptoms ever. The total prevalence of each indicator equals the sum of its unique prevalence and its overlapping prevalence

Venn diagrams of the prevalence and overlapping of the several disease indicators are shown in Fig. 1. Indicators of current and ever disease show a very similar pattern, with rhinoconjunctivitis indicators being the most frequent ones and eczema indicators following. The overlapping of the 3 disease indicators for current and ever symptoms was quite substantial with about one-third of asthmatics having either rhinoconjunctivitis or eczema. A very similar pattern arose for eczema sufferers. Not many individuals with rhinoconjunctivitis symptoms had also symptoms of eczema or asthma or both. For severe symptoms, the picture is quite different: the overlapping is almost absent and the prevalence of severe symptoms of asthma is slightly higher than that of severe rhinoconjunctivitis (Fig. 1).

Table 2 show the results of the univariate logistic regression analysis for factors which associations with the different disease indicators were significant on at least 1 category. Tables 3 and 4 depict the associations obtained by the 2 multivariate logistic regressions: the adjusted and the fully adjusted models. Table 1, Table 2, Table 3 show a quite consistent pattern of associations. Vigorous exercise was associated with higher prevalence of most disease indicators, including (albeit to a lesser extent) eczema. Consumption of paracetamol at least 1 per month in the last 12 months was associated to indicators of current rhinoconjunctivitis and hay fever being the corresponding faORs in the fully adjusted model: faOR 2.22 (95%CI 1.41–3.48) and faOR 1.90 (95%CI 1.28–2.84). The corresponding figure for current eczema indicator was faOR 2.82 (1.18–6.71). There was also significant association with asthma ever in the adjusted model (faOR 2.67; 95%CI 1.03–6.93) whose strength was somewhat reduced in the fully adjusted model (aOR 2.16; 95%CI 0.83–5.66). Only olive oil and burger intake showed any association with disease indicators. Olive oil consumption most of all days was associated with a lower prevalence of indicators of current asthma (faOR 2.82; 95%CI 1.18–6.71) and asthma ever (faOR 0.48; 95%CI 0.24–0.99). There was also a trend of this type of association with eczema ever indicator (aOR 0.60; 95%CI 0.36–1.00; and faOR 0.61; 95%CI 0.37–1.01). Conversely, burger intake most of all days was associated to higher prevalence of current rhinoconjunctivitis indicator (faOR 1.65; 95%CI 1.07–2.56). BMI was significantly associated with current rhinoconjunctivitis indicator (faOR 1.05; 95%CI 1.00–1.10) (see Table 4).

**Table 2:** Crude associations expressed as ORs and 95%CI between disease indicators and potential risk/protective factors included in the GAN environmental questionnaire (only shown those p < 0.15 in at least 1 of the categories).

	Current asthma	Severe asthma	Asthma ever	Current rhinoconjunctivitis	Severe rhinoconjunctivitis	Hay fever ever	Current eczema	Severe eczema	Eczema ever
Male sex	N.A.	N.A.	N.A.	0.69 (0.55–0.86)	N.A.	0.77 (0.62–0.94)	0.58 (0.40–0.83)	0.38 (0.16–0.94)	0.51 (0.35–0.76)
Exercise:									
Never/Occasionally	1	1	1	1	1	1	1	1	1
Once/twice per week	**3.50 (2.19–5.63)**	**5.17 (2.71–9.89)**	**2.01 (1.68–3.47)**	**1.92 (1.46–2.50)**	**2.81 (1.38–5.74)**	1.01 (0.77–1.34)	**2.26 (1.52–3.37)**	1.08 (0.36–3.26)	**1.78 (1.16–2.72)**
Three + times per week	2.14 (0.99–4.64)	**3.78 (1.47–9.71)**	**3.20 (1.65–6.21)**	**1.78 (1.20–2.66)**	**6.38 (2.98**–**13**–**62)**	1.36 (0.93–2.00)	**2.03 (1.12–3.67)**	2.82 (0.93–8.61)	0.83 (0.36–1.93)
Truck traffic in the home street:									
Never	N.A.	N.A.	N.A.	N.A.	N.A.	N.A.	1	1	1
Seldom (not often)							1.36 (0.61–3.05)	1.78 (0.22–14.08)	1.76 (0.69–4.48)
Frequently through the day							**2.30 (1.02–5.20)**	1.56 (0.17–14.00)	**3.07 (1.20–7.90)**
Almost the whole day							**2.77 (1.21–6.35)**	4.54 (0.56–36.51)	**3.15 (1.20–8.29)**
Paracetamol intake last year:									
Never	1	1	1	1	1	1	1	1	1
Al least once per year	0.89 (0.43–1.84)	0.79 (0.31–2.04)	1.28 (0.56–2.94)	**1.50 (1.00–2.24)**	0.77 (0.27–2.18)	**1.86 (1.29–2.69)**	**3.13 (1.34–7.29)**	3.60 (0.47–27.76	0.87 (0.48–1.58)
At least once per month	1.92 (0.96–3.87)	1.81 (0.75–4.47)	**2.49 (1.10–5.64)**	**2.74 (1.83–4.11)**	2.18 (0.82–5.75)	**2.16 (1.48–3.16)**	**4.64 (1.99–10.8)**	4.51 (0.57–35.39)	1.55 (0.86–2.80)
Olive oil intake:									
Never or only occasionally	1	1	1	1	1	N.A.	N.A.	N.A.	1
Once or twice per week	1.02 (0.51–2.04)	1.53 (0.64–3.66)	0.74 (0.35–1.58)	0.70 (0.48–1.01)	0.77 (0.55–1.08)				0.55 (0.29–1.04)
Most or all days	**0.21 (0.06–0.76)**	0.32 (0.08–1.26)	0.39 (0.14–1.07)	**0.56 (0.36–0.87)**	**0.62 (0.41–0.92)**				**0.47 (0.22–0.99)**
Burger intake:									
Never or only occasionally	1	N.A.	N.A.	1	N.A.	N.A.	N.A.	N.A.	N.A.
Once or twice per week	1.21 (0.50–2.97)			1.24 (0.79–1.95)					
Most or all days	**7.93 (1.42–44.2)**			2.03 (0.89–4.66)					
Body Mass Index (per unit)	N.A.	1.10 (0.99–1.22)	N.A.	1.03 (0.99–1.08)	N.A.	N.A.	N.A.	N.A.	1.05 (0.98–1.13)

N.A.: No association (p > 0.15).

Regular fonts: Marginal associations (0.05 < p < 0.15).

Bold fonts: Significant associations (p < 0.05)

**Table 3:** Adjusted^a^ associations expressed as ORs and 95%CI between disease indicators and potential risk/protective factors included in the GAN environmental questionnaire.

	Current	Severe	Asthma	Current	Severe	Hay fever	Current	Severe	Eczema
	asthma	asthma	ever	rhinoconjunctivitis	rhinoconjunctivitis	ever	eczema	eczema	ever
Male sex	N.I..	N.I..	N.I..	0.78 (0.60–1.01)	N.I..	0.80 (0.64–0.99)	0.65 (0.45–0.96)	0.39 (0.15–1.00)	0.60 (0.39–0.92)
Exercise:									
Never/Occasionally	1	1	1	1	1	1	1	1	1
Once/twice per week	**3.33 (1.99–5.57)**	**4.72 (2.41–9.26)**	**1.79 (1.00–3.19)**	**1.74 (1.30–2.33)**	**2.55 (1.20–5.42)**	0.99 (0.74–1.31)	**1.99 (1.31–3.01)**	1.04 (0.33–3.22)	1.41 (0.89–2.24)
Three + times per week	**2.28 (1.03–5.02)**	**3.55 (1.36–9.25)**	**3.15 (1.60–6.19)**	**1.81 (1.76–2.79)**	**5.81 (2.60–12.98)**	1.36 (0.91–2.03)	**2.12 (1.15–3.94)**	**3.65 (1.11–12.01)**	0.86 (0.36–2.06)
Truck traffic in the home street:									
Never	N.I.	N.I.	N.I.	N.I.	N.I.	N.I.	1	1	1
Seldom (not often)							1.09 (0.48–2.46)	1.47 (0.18–11.75)	2.64 (0.80–8.66)
Frequently through the day							1.59 (0.69–3.66)	1.08 (0.12–9.97)	**3.82 (1.15–12.72)**
Almost the whole day							2.02 (0.87–4.70)	3.33 (0.41–27.33)	**4.38 (1.29–14.88)**
Paracetamol intake last year:									
Never	1	1	1	1	1	1	1	1	1
Al least once per year	0.94 (0.42–2.10)	0.78 (0.28–2.16)	1.46 (0.56–3.82)	1.41 (0.91–2.20)	0.76 (0.24–2.39)	**1.81 (1.24–2.65)**	**2.59 (1.10–6.09)**	2.64 (0.34–20.75)	0.73 (0.39–1.40)
At least once per month	1.64 (0.74–3.60)	1.49 (0.55–4.03)	**2.67 (1.03–6.93)**	**2.41 (1.54–3.77)**	2.14 (0.73–6.30)	**2.02 (1.36–2.99)**	**3.54 (1.50–8.3**6)	3.19 (0.40–25.59)	1.22 (0.64–2.33)
Olive oil intake:									
Never or only occasionally	1	1	1	1	1	N.I.	N.I.	N.I.	1
Once or twice per week	0.89 (0.52–1.53)	1.43 (0.74–2.74)	0.89 (0.51–1.54)	0.85 (0.63–1.14)	0.63 (0.29–1.39)				**0.50 (0.30–0.84)**
Most or all days	**0.37 (0.18–0.79)**	0.36 (0.12–1.06)	**0.47 (0.23–0.96)**	0.78 (0.57–1.07)	0.65 (0.28–1.47)				**0.60 (0.36–1.00)**
Burger intake:									
Never or only occasionally	1	N.I.	N.I.	1	N.I.	N.I.	N.I.	N.I.	N.I.
Once or twice per week	1.17 (0.69–1.97)			1.25 (0.95–1.64)					
Most or all days	1.51 (0.66–3.47)			**1.70 (1.10–2.61)**					
Body Mass Index (per unit)	N.I.	1.11 (0.99–1.23)	N.I.	**1.05 (1.00–1.10)**	N.I.	N.I.	N.I.	N.I.	1.06 (0.99–1.14)

N.I..: Not included in the multivariate logistic regression as no indication of association was found in the bivariate analysis.

Regular fonts: factors included in the multivariate analysis due to marginal (p < 0.15) or significant associations (p < 0.05) found in the bivariate analysis.

Bold fonts: Significant associations (p < 0.05).

a Adjusted for all factors included in the table

**Table 4:** Fully adjusted^a^ associations expressed as ORs and 95%CI between disease indicators and potential risk/protective factors included in the GAN environmental question-naire.

	Current	Severe	Asthma	Current	Severe	Hay fever	Current	Severe	Eczema
	asthma	asthma	ever	rhinoconjunctivitis	rhinoconjunctivitis	ever	eczema	eczema	ever
Male sex	N.I.	N.I.	N.I.	0.81 (0.63–1.05)	N.I.	0.82 (0.66–1.03)	0.70 (0.48–1.04)	0.37 (0.14–0.97)	0.62 (0.40–0.95)
Exercise:									
Never/Occasionally	1	1	1	1	1	1	1	1	1
Once/twice per week	**2.86 (1.69–4.86)**	**4.57 (2.33–9.00)**	**1.82 (1.01–3.28)**	**1.53 (1.13–2.07)**	**2.38 (1.11–5.12)**	0.91 (0.68–1.22)	**1.63 (1.06–2.52)**	1.00 (0.32–3.14)	1.43 (0.90–2.28)
Three + times per week	1.98 (0.88–4.41)	**3.33 (1.27–8.78)**	**2.98 (1.49–5.95)**	**1.64 (1.05–2.56)**	**5.50 (2.44–12.38)**	1.24 (0.83–1.87)	1.80 (0.96–3.39)	3.28 (0.95–11.31)	0.82 (0.34–1.98)
Truck traffic in the home street:									
Never	N.I.	N.I.	N.I.	N.I.	N.I.	N.I.	1	1	1
Seldom (not often)							1.11 (0.49–2.52)	1.43 (0.18–11.47)	2.58 (0.79–8.49)
Frequently through the day							1.54 (0.66–3.58)	0.99 (0.11–9.25)	**3.66 (1.10–12.20)**
Almost the whole day							1.99 (0.84–4.68)	3.06 (0.37–25.31)	**4.08 (1.20–13.92)**
Paracetamol intake last year:									
Never	1	1	1	1	1	1	1	1	1
Al least once per year	0.84 (0.37–1.88)	0.78 (0.28–2.17)	1.21 (0.46–3.19)	1.36 (0.87–2.13)2	0.74 (0.23–2.33)	**1.83 (1.25–2.68)**	**2.43 (1.03–5.72)**	2.66 (0.34–20.91)	0.67 (0.35–1.28)
At least once per month	1.28 (0.58–2.86)	1.43 (0.53–3.89)	2.16 (0.83–5.66)	**2.22 (1.41–3.48**)	2.01 (0.68–5.92)	**1.90 (1.28–2.84)**	**2.82 (1.18–6.71)**	3.07 (0.38–24.65)	1.11 (0.58–2.12)
Olive oil intake:									
Never or only occasionally	1	1	1	1	1	N.I.	N.I.	N.I.	1
Once or twice per week	0.90 (0.52–1.56)	1.45 (0.76–2.80)	0.93 (0.53–1.63)	0.85 (0.63–1.16)	0.62 (0.28–1.37)				**0.50 (0.30–0.85)**
Most or all days	**0.38 (0.18–0.81)**	0.36 (0.12–1.07)	**0.48 (0.24–0.99)**	0.79 (0.58–1.09)	0.66 (0.29–1.50)				0.61 (0.37–1.01)
Burger intake:									
Never or only occasionally	1	N.I.	N.I.	1	N.I.	N.I.	N.I.	N.I.	N.I.
Once or twice per week	1.15 (0.68–1.95)			1.27 (0.96–1.67)					
Most or all days	1.29 (0.55–3.04)			**1.65 (1.07–2.56)**					
Body Mass Index (per unit)	N.I.	1.10 (0.99–1.23)	N.I.	**1.05 (1.00–1.10)**	N.I.	N.I.	N.I.	N.I.	1.06 (0.99–1.14)

plus current rhinoconjunctivitis and current eczema).

N.I.: Not included in the multivariate logistic regression analysis as no indication of association was found in the bivariate analysis.

Regular fonts: factors included in the multivariate analysis due to marginal (p < 0.15) or significant associations (p < 0.05) found in the bivariate analysis.

Bold fonts: Significant associations (p < 0.05).

a Adjusted for all factors included in the table and for the corresponding disease indicators not included as dependent variable (for instance, associations of factors with current asthma include all factors in the table

## Discussion

The present study, a survey following the GAN methodology in a quite extreme environment, has found a low prevalence of allergic disease indicators which is consistent with previous findings in the ISAAC and GAN general reports^11^, ^12^, ^13^ and specifically in those from centre in high altitudes.^14^^,^^15^

### Comparison with the prevalence of asthma, rhinoconjunctivitis, and eczema in high-altitude populations

We found a prevalence of current asthma of 3.1% (Table 1) which is comparable with the prevalence of 2.6% in the Shigatze District of Tibet (altitude 3900 m.a.s.l) 2 decades ago in a population of adolescents 12–14 years of age.^15^ Similar results were found around the same time (both studies were carried out under the ISAAC Phase Three survey collaboration^16^) in Tibetan Lhasa Valley, where current asthma prevalence was found to be 0.7%, 0.5% and 2.8% respectively for Tibetan, Han and Hui ethnicities.^14^

According to a recent systematic review, most asthma prevalence studies in high altitudes have been carried out within the ISAAC collaboration and consistently found that high altitudes (including centres above 1500 m.a.s.l) were related to lower prevalence of asthma.^17^, ^18^, ^19^, ^20^ An altitude of 1500 m.a.s.l. above sea level has been shown to be a threshold above which asthma prevalence is highly and negatively correlated with altitude.^21^ However, depending on the country, allergic diseases can be quite prevalent even at high altitudes (>2000 m.a.s.l.) as in the case of Costa Rica where Soto-Quirós et al found a prevalence of current asthma (as per ISAAC definition) of asthma of 27.9%. In the same continent and in similar altitudes, using the same methods, asthma prevalences range between Tibet and Costa Rica, as it occurred in Mexico City (3.9%–10.0% depending on the district), Bogota, Colombia (8.5%) or Calama, Chile (11.1%). Although ISAAC Phase Three clearly showed that altitude (probably as a marker of other factors such as climate, allergens or lifestyle) is a statistically significant protective factor for asthma, it could only explain 15% of worldwide variability,^20^^,^^22^ which means that other factors, such as lifestyle, ethnicity or other environmental conditions, play a much more important role.

Current rhinoconjunctivitis in Naryn had a much lower prevalence (13.3%) than centres elsewhere in Asia, such as those in Iran (Karaj, 34.2%; Yazd, 36.4%) and Syria (Damascus, 39.8%; Lattakia, 47.1%). In fact, only 1 centre in GAN Phase I (Lucknow, India) reported a lower prevalence (12.5%).^1^ Still Tibet showed even lower prevalences ranging 3.2%–12.5%.^14^^,^^15^

Contrary to asthma, rhinoconjunctivitis did not show any association with altitude in the ISAAC study, neither in the adolescents’ group nor in the 6-7 years-old children.^20^ This was not the case in a recent study non-ISAAC/GAN study, albeit in younger children (6–7 years), which found higher prevalence of rhinitis (as defined by a questionnaire specifically designed for the study) in the Chinese province of Yunnan, comparing the prefecture of Xishuangbanna (low altitude) with that of Diqing (high altitude). A more recent GAN study in Mexico also found a trend toward a lower prevalence at higher altitude (>1500 m.a.s.l.), but only among female adolescents.^23^ However, as in asthma, the prevalence of rhinoconjunctivitis was lower in GAN low-altitude centres in India than in high-altitude centres in Mexico (for example, 12.5% in Lucknow, India, vs. 22.9% in rural Toluca, Mexico).^1^

Current eczema prevalence was found to be quite variable in centres around Kirzgyzstan, ranging from 2.9% in Yazd (Iran) and 10.2% in Lattakia (Syria) as reported in the GAN study recently.^1^ In high altitude centres in Mexico (2000+ m.a.s.l.) the prevalence of this condition tended to be lower in centres in higher altitude. In fact, there was an association between altitudes below 1000 m.a.s.l. and higher prevalence^24^ in this country. However, the lowest prevalence rates in GAN survey were found in Mysore, India (1,4%) (745 m.a.s.l.) and in Anuradhapura, Sri Lanka (1.1%) (80 m.a.s.l.). Overall, these data suggests that altitude (and all related climate factors) has a very modest role in the variability of the prevalence of current eczema worldwide.

### Comparison with previous surveys in Kyrgyzstan

The first published data on allergy prevalence among adolescents in Kyrgyzstan were collected as part of the ISAAC study in 2001 in Jalal-Abad, Bishkek and Balykchy. Jalal-Abad (760 m.a.s.l.) has a warm and dry climate with high summer temperatures, whereas Balykchy (1900 m.a.s.l.) and Naryn (2066 m.a.s.l) have continental climate, characterized by cold, long winters and warm, dry summers. Bishkek (700 m.a.s.l) has a Mediterranean-influenced humid continental climate. These climatic differences likely translate into distinct living conditions, lifestyles, and dietary patterns in the 2 regions. Therefore, comparisons between the 2 cities should be interpreted with caution.

In ISAAC Phase Three, the prevalence of current asthma^12^ in Bishkek, Balykchy, and Jalal-Abad was 8.0%, 7.2%, and 7.7%, respectively. For current rhinoconjunctivitis,^11^ the corresponding prevalences were 8.3%, 9.1%, and 13.8%; and for current eczema,^5^^,^^13^ 2.9%, 2.8%, and 4.9%.

In a more recent survey (also using the ISAAC methods), adolescents in Jalal-Abad showed the following prevalences of current allergic symptoms: asthma, 13.7%; rhinoconjunctivitis, 16.6%; and eczema, 10.0%.^4^ These prevalences differ both from those observed recently in Naryn and from those in Jalal-Abad 20 years ago. Overall, they are substantially higher, particularly for asthma. Differences in survey time points and, more importantly, differences in ethnicity, climate, altitude and associated environmental exposures may account for the observed differences.

### Risk factors and protective factors

Consistent factors associated with at least 1 indicator of asthma, rhinoconjunctivitis, or eczema in the present survey were increased physical activity, frequent paracetamol use, and low olive oil intake. Higher burger intake and higher BMI were associated only with current rhinoconjunctivitis. Truck traffic was associated only with eczema ever. The number of smokers was too low to detect any association.

The association between current eczema and increased paracetamol intake is of interest because, as previously shown, both at the individual and school-level in both affluent and non-affluent countries, confounding by indication is unlikely.^25^^,^^26^ Fast food consumption has been also shown to be a risk factor for rhinoconjunctivitis within the ISAAC study also in affluent and non-affluent countyries.^26^^,^^27^

There is limited information on the effects of olive oil consumption on eczema, although some evidence suggests that adherence to a Mediterranean diet supplemented with extra-virgin olive oil during pregnancy may reduce the risk of infant eczema in offspring.^28^

Interestingly, BMI was associated with rhinoconjunctivitis but showed no association with asthma (aside from a marginal association with severe asthma) or eczema. The association between BMI and rhinoconjunctivitis has shown variable and inconsistent results, according to a recent metanalysis.^29^

The most consistent finding in the present survey was the association of physical activity with all allergy indicators. While the associations with asthma and rhinoconjunctivitis may be explained, at least in part, by exercise-induced symptoms, this interpretation is less straightforward for current eczema. Two recent GAN surveys in Saudi Arabia^30^ and Kosovo^31^ have also shown this association in adolescents, which had been reported previously in Lebanon^32^ and in the global ISAAC anslysis.^33^ It does not appear that this association depends on asthma or rhinoconjunctivitis, as it remains essentially unchanged even when both conditions are included in the analysis (Table 2 vs Table 3). This pattern was also observed in the Kosovo study, which performed a stratified analysis by asthma symptoms and found that the association persisted in the subgroup of non-asthmatic adolescents..^31^ It is difficult to determine whether this association is attributable to sweating during exercise or to skin exposure to environmental or climatic conditions.

### Strengths and limitations

As any cross-sectional study the present one cannot infer causal relationship but only associations. Although the definitions of asthma, rhinoconjunctivitis and eczema has been widely used in epidemiological studies, they may not capture the entire clinical diagnosis and thus classification bias cannot be ruled out. Because both exposure and outcome are self-reported, some random misclassification is possible. This would be expected to introduce noise and most often attenuate the observed association; therefore, in a scenario with more accurate classification, the true association might be stronger. A large sample size should help limit the impact of this random misclassification on precision, although some attenuation may remain. Finally, using standardized definitions facilitates comparisons with other centres and countries.

### Summary

The present study shows that, according to the GAN Phase I survey,^1^ the prevalence of indicators of asthma, rhinoconjunctivitis, and eczema in Kyrgyzstan is among the lowest worldwide. It also identifies positive and negative associations for some of these conditions that have been reported previously. In particular, the positive association between exercise and most allergy indicators, and the inverse association between olive oil intake and asthma, merit emphasis. Paracetamol use and burger consumption were also positively associated with several indicators.

## Authors’ contributions

• Study design: LGM is part of the Steering Committee of the Global Asthma Network, and participated in the survey design.
• Data acquisition**:** SAS, MEA, AAM, NNB, ZKM, SEO, KTT and BEN.
• Dataset cleaning and checking: LGM, AMT, SAS and ZKM.
• Statistical analyses: LGM.
• Manuscript drafting: LGM and AMT.
• Manuscript reviewing and approval: All authors.

## Ethics approval and consent to participate

Before starting the study, approval was obtained from the local ethics committee. An application was submitted to the school, containing the study protocol, participant safety information, and information about adolescents’ passive consent; before answering the questionnaire, adolescents were advised that their participation was voluntary.

## Consent for publication

Not applicable.

## Availability of data and material

Data are available from the corresponding author by request.

## Declaration of generative AI and AI-assisted technologies in the writing process

AI was used to check the grammar correctness of the final manuscript in order to improve readability and language.

## Funding

The GAN data centre at the 10.13039/501100004687University of Murcia was funded by 10.13039/501100004587Instituto de Salud Carlos III, reference PI17/00179.

## Declaration of competing interest

The authors declare no conflict of interest.
