# Supplementary material for: Prevalence and risk factors of asthma, rhinoconjunctivitis and eczema in the very extreme environment of Naryn, Kyrgyzstan
Source: World Allergy Organ J. 2026 May 1;19(5):101386. doi: 10.1016/j.waojou.2026.101386 (PMC13145855; doi:10.1016/j.waojou.2026.101386)
Supplement: Multimedia component 1 [file mmc1.docx]

Web Table 1. Potential risk or protective factors included in the study.

|  | N | % |
| --- | --- | --- |
| Male sex | 1416 | 53.0 |
| Exercise:   - Never/Occasionally - Once/twice per week - Three + times per week - Missing | 1902  471  182  118 | 71.2  17.6  6.8  4.4 |
| Television watching/week:   - less than 1 hour, - 1 hour but less than 3 hours - 3 hours but less than 5 hours - 5 hours or more - Missing | 1257  777  330  230  79 | 47.0  29.1  12.4  8.6  3.0 |
| Computer use/week:   - less than 1 hour, - 1 hour but less than 3 hours - 3 hours but less than 5 hours - 5 hours or more - Missing | 277  567  651  1115  63 | 10.4  21.2  24.4  41.7  2.4 |
| Truck traffic in the home street:   - Never - Seldom (not often) - Frequently through the day - Almost the whole day - Missing | 253  1286  651  452  31 | 9.5  48.1  24.4  16.9  1.2 |
| Paracetamol intake last year:   - Never - Al least once per year - At least once per month - Missing | 392  1316  876  89 | 14.7  49.2  32.8  3.3 |
| Cat ownership   - Yes - No - Missing | 991  1617  65 | 37.1  60.5  2.4 |
| Dog ownership   - Yes - No - Missing | 782  1801  90 | 29.3  67.4  3.4 |
| Ever smoker   - Not at all - Less than daily - Daily - Missing | 2358  163  15  137 | 88.2  6.1  0.6  5.1 |
| Current smoker   - Not at all - Less than daily - Daily - Missing | 2338  61  9  265 | 87.5  2.3  0.3  9.9 |
| Water pipe smoking  - Yes  - No  - Missing | 12  2410  251 | 0.5  90.2  9.4 |
|  | Median | Interquartile  range |
| Old siblings | 1 | 0-2 |
| Young siblings | 2 | 1-2 |
| Age at starting smoking | 12 | 9-13 |
| Number of cigarettes per day | 1 | 1-2 |
| Body Mass Index | 18.4 | 17.0-20.0 |
